# ADAM8 is expressed widely in breast cancer and predicts poor outcome in hormone receptor positive, HER-2 negative patients

**DOI:** 10.1186/s12935-023-03024-3

**Published:** 2023-08-11

**Authors:** Stefania Pianetti, Kathy D. Miller, Hannah H. Chen, Sandra Althouse, Sha Cao, Steven J. Michael, Gail E. Sonenshein, Nora D. Mineva

**Affiliations:** 1 Department of Developmental, Molecular, and Chemical Biology, Tufts University School of Medicine, 136 Harrison Ave., Boston, MA 02111 USA; 2 Adecto Pharmaceuticals, Inc., 75 Kneeland St., 14th Floor, Boston, MA 02111 USA; 3 Indiana University Melvin and Bren Simon Comprehensive Cancer Center, Indianapolis, IN USA; 4 Department of Pathology and Laboratory Medicine, Tufts Medical Center, Boston, MA USA; 5 Department of Biostatistics and Health Data Science, Indiana University School of Medicine, Indianapolis, IN USA

**Keywords:** ADAM8, Breast cancer, Biomarker, Immunohistochemistry, Diagnosis, Prognosis

## Abstract

**Background:**

Breast malignancies are the predominant cancer-related cause of death in women. New methods of diagnosis, prognosis and treatment are necessary. Previously, we identified the breast cancer cell surface protein ADAM8 as a marker of poor survival, and a driver of Triple-Negative Breast Cancer (TNBC) growth and spread. Immunohistochemistry (IHC) with a research-only anti-ADAM8 antibody revealed 34.0% of TNBCs (17/50) expressed ADAM8. To identify those patients who could benefit from future ADAM8-based interventions, new clinical tests are needed. Here, we report on the preclinical development of a highly specific IHC assay for detection of ADAM8-positive breast tumors.

**Methods:**

Formalin-fixed paraffin-embedded sections of ADAM8-positive breast cell lines and patient-derived xenograft tumors were used in IHC to identify a lead antibody, appropriate staining conditions and controls. Patient breast cancer samples (n = 490) were used to validate the assay. Cox proportional hazards models assessed association between survival and ADAM8 expression.

**Results:**

ADAM8 staining conditions were optimized, a lead anti-human ADAM8 monoclonal IHC antibody (ADP2) identified, and a breast staining/scoring control cell line microarray (CCM) generated expressing a range of ADAM8 levels. Assay specificity, reproducibility, and appropriateness of the CCM for scoring tumor samples were demonstrated. Consistent with earlier findings, 36.1% (22/61) of patient TNBCs expressed ADAM8. Overall, 33.9% (166/490) of the breast cancer population was ADAM8-positive, including Hormone Receptor (HR) and Human Epidermal Growth Factor Receptor-2 (HER2) positive cancers, which were tested for the first time. For the most prevalent HR-positive/HER2-negative subtype, high ADAM8 expression identified patients at risk of poor survival.

**Conclusions:**

Our studies show ADAM8 is widely expressed in breast cancer and provide support for both a diagnostic and prognostic value of the ADP2 IHC assay. As ADAM8 has been implicated in multiple solid malignancies, continued development of this assay may have broad impact on cancer management.

## Background

Breast cancer is the primary cause of cancer deaths in women globally [700,000 yearly, World Health Organization (WHO)], mainly from metastatic disease. Breast cancers that express the hormone receptors (HRs) Estrogen Receptor-α (ER) and Progesterone Receptor (PR), but not Human Epidermal Growth Factor Receptor-2 (HER2) [HR+/HER2−] are the most common subtype (68%), followed by HR+/HER2+ and HR−/HER2− (10% each) and HR−/HER2+ (4%) [NCI]. Endocrine therapies, e.g., the selective estrogen receptor modulator (SERM) Tamoxifen, block estrogen signaling in HR+ breast cancer cells, decreasing recurrence and improving survival [1]. The anti-HER2 antibody Trastuzumab, the first monoclonal antibody approved for the treatment of a solid tumor, revolutionized treatment of HER2+  breast cancer [1]. Now, various HER2-targeted therapies, in addition to Trastuzumab, can be given to improve outcome, for example, the complementary anti-HER2 targeting antibody Pertuzumab or the antibody–drug conjugate Ado-trastuzumab emtansine [1]. While patients with HR-driven and/or HER2-driven breast cancer have benefited enormously from such endocrine and HER2-targeted therapies, unfortunately, these tumors still account for the majority of breast cancer deaths. The HR−/HER2− or ER−/PR−/HER2− subtype, also known as Triple-Negative Breast Cancer (TNBC) occurs preferentially in younger women and in women of African-American descent, and is even more challenging to treat due to a lack of ER, PR or HER2 target expression.

ADAM8 is a transmembrane, cell surface protein member of the ADAM (A Disintegrin And Metalloprotease) family that mediates cell adhesion and migration, as well as proteolysis of various substrates, including cell adhesion molecules, cytokine receptors or ligands, and components of the extracellular matrix [2]. Previously, knockdown and overexpression studies in breast cancer cells demonstrated ADAM8 promotes cell migration, invasion through Matrigel and growth in an anchorage independent fashion, while having little effect on cell proliferation [3]. Furthermore, knockdown and antibody targeting strategies in TNBC orthotopic mouse models showed ADAM8 promotes both tumor growth and spread [3]. The ADAM8 Metalloproteinase (MP) domain promoted release of various factors (e.g., VEGF-A, PDGF-AA, angiogenin) from the tumor cell surface that mediate angiogenesis and tumor growth [3]. The ADAM8 Disintegrin (DI) domain mediated TNBC cell adhesion to endothelial cells via activation of integrins on the cancer cell surface that permits intravasation through the blood vessel wall into the blood stream as well as extravasation at distant sites to establish metastases [3]. Pooling of RNA microarray studies from Oncomine demonstrated *ADAM8* is one of the more highly expressed genes in breast cancer vs normal tissue (P = 0.025), and that high *ADAM8* mRNA levels significantly correlate with poor disease-free survival (DFS) and overall survival (OS) in Kaplan–Meier analyses of the total patient population [3]. Similarly, high ADAM8 expression has been detected in multiple other solid tumors, i.e., lung, liver, pancreas, stomach, colon, bone, head and neck, and associated with either poorer prognosis, more metastatic phenotype or higher tumor grade [4–10].

Of note, ADAM8 is an ideal target for therapeutic intervention as it has been demonstrated to be non-essential under physiological conditions, that is, ADAM8 deficient mice develop normally, free of pathological defects, and have a normal lifespan [11, 12]. In immunohistochemistry (IHC), using a commercial research-only antibody, ADAM8 was abundantly expressed in 34.0% (17/50) of TNBC biopsies, whereas adjacent histologically normal breast tissue was negative (0/50) [3]. Consistently, we find limited ADAM8 expression in an FDA Standard Normal Human Tissue Array (unpublished findings). We have recently isolated and characterized a panel of extremely specific mouse monoclonal dual MP and DI antagonist anti-ADAM8 antibodies (mAbs), termed ADPs, for use in therapy of TNBC patients (manuscript in preparation). To identify patients who could benefit from an anti-ADAM8 therapeutic, here, we report on the preclinical development of a diagnostic IHC assay based on the highly specific ADP mAbs and the identification of ADP2 as lead. To validate the ability of this assay to detect ADAM8 expression in tissue samples, a set of tissue microarrays (TMAs) with 490 breast cancer biopsies, including 412 with data on expression of ER, PR and HER2 (including 61 TNBCs across the panel), were analyzed. ADAM8 expression was confirmed in 36.1% (22/61) of TNBCs, consistent with our earlier studies using a research-only antibody [3]. The analysis revealed a similar positivity rate (~ 30%) in the non-TNBC breast cancer subtypes, including HR+/HER2−, HR−/HER2+, and HR+/HER2+ that had not been examined previously. Of note, a 10-year age and race adjusted Cox proportional hazards model for the HR+/HER2− subtype, with the largest sample size, revealed high ADAM8 expression in these patients was associated with poorer survival, suggesting a potential prognostic value for this assay in addition to its intended companion diagnostic purpose.

## Methods

### Patients, specimen characteristics and TMA construction

Breast cancer samples and patient medical records, including demographics, pathology, treatment, and follow-up information were collected for > 600 cases between 1989 and 2005 at Indiana University Health (University and Methodist Hospitals, Indianapolis, IN, USA). Samples and patient information were obtained under an Indiana University Institutional Review Board (IRB) protocol with waiver of informed consent as only de-identified data are associated with the TMA and available to researchers. Fourteen TMAs, containing 577 primary breast tumors from these archival cases (in duplicate cores) were constructed at the Tissue Procurement and Distribution Core at the Indiana University Melvin and Bren Simon Comprehensive Cancer Center (Indianapolis, IN, USA). Freshly cut TMA slides were subjected to H&E for tissue quality control and IHC for ADAM8 expression. Following exclusion of samples that had artifacts/defects or that were depleted, a total of 490 unique cases, including 412 biopsies with data on the expression of all 3 standard molecular markers for breast cancer (ER, PR and HER2), were available for analysis. Samples with any breast cancer histology were included. Patients ranged between 28 and 94 years of age; 489 were assigned female at birth while one was male. Sample ER and PR status was determined using IHC at time of diagnosis and reported as positive or negative. HER2 status was determined using IHC at time of diagnosis or retrospectively and reported on a standard 0–3 scale, where 0 and 1 scoring samples are considered negative, 2 equivocal and 3 positive. When available, fluorescence in situ hybridization (FISH) testing was used to determine whether HER2 equivocal samples are positive or negative. Samples with missing ER, PR or HER2 marker status, or those with equivocal HER2 IHC staining, and no FISH data were considered to have an unknown status and excluded from subtype analyses, but kept in studies of the total breast cancer population.

### Cell lines

Human, non-tumoral, ER-, MCF-10A mammary epithelial cells and human TNBC MDA-MB-231 breast cancer cells were purchased from American Type Culture Collection (ATCC, Manassas, VA, USA) and maintained as recommended by the ATCC. The triple-negative inflammatory human breast cancer line SUM149, which was graciously provided by Stephen Ethier (Medical University of South Carolina, Charleston, SC, USA), was maintained as published [13]. Human embryonic kidney (HEK)-293 cells were purchased from ATCC and maintained in DMEM supplemented with 10% FBS. Stable clones of HEK-293 cells expressing human ADAM8 (HEK-A8) or control empty vector DNA (HEK-EV) were prepared by transfection of 1 μg of either pCMV6-human ADAM8 Variant 1-AC-GFP plasmid (RG213386, Origene, Rockville, MD, USA) or control pCMV6-AC-GFP DNA (PS100010, Origene, Rockville, MD, USA), respectively, using Lipofectoamine 2000 (11668019, Invitrogen, Waltham, MA), followed by selection in 500 μg/ml G418. Short tandem repeat analysis was used to authenticate cell lines (Labcorp, Cincinnati, OH, USA). For 2D culture, Accutase (A1110501, Thermo Fisher Scientific, Waltham, MA, USA) was used to dissociate confluent 100 mm plates and cells subcultured at dilutions of 1:3 (MCF10A-2D), 1:2 (MDA-MB-231-2D) and 1:5 (HEK-A8-2D and HEK-EV-2D) onto tissue culture 100 mm plates and grown to confluency (48–72 h). For 3D culture, confluent 100 mm plates of 2D grown MDA-MB-231 cells were dissociated as above, and the resulting single cell suspension plated on a 100 mm low attachment plate (664970, Greiner Bio-One, Monroe, NC, USA) and cultured for 48 h.

### Immunoblotting

Whole cell extracts (WCE) were subjected to Western blotting for ADAM8 as previously described [3]. Briefly, 2D and 3D cell cultures were collected using scraping or centrifugation, respectively, washed in 1XPBS and exposed to Radioimmunoprecipitation assay buffer (RIPA, 50 mM Tris pH 7.6, 150 mM NaCl, 1% NP40, 0.1% SDS, 5 mM EDTA, 1% Sodium Sarkosyl) supplemented with Halt Protease and Phosphatase Inhibitor Single-Use Cocktail (1:100, 78442, Thermo Fisher Scientific, Waltham, MA, USA), 0.5 M EDTA (1:100) and 1 M 1,10-Phenanthroline (1:100, 131377, MilliporeSigma, Burlington, MA, USA) to inhibit the autocatalytic activity of ADAM8. Lysates were sonicated and centrifuged at 16,000×*g* for 15 min. Protein concentrations were calculated using a DC Protein Assay Reagent (Bio-Rad Laboratories, Hercules, CA, USA). Samples (30 μg) were separated in 8% polyacrylamide-SDS gels and analyzed by Immunoblotting for ADAM8 with the LS-C20181 anti-ADAM8 antibody (LifeSpan BioSciences, Seattle, WA, USA). Molecular mass markers were included on each gel (1610394, Bio-Rad Laboratories, Hercules, CA, USA). Blotting for β-actin (A5441, MilliporeSigma, Burlington, MA, USA) was used to control for equal loading.

### Control cell line microarray

Cultures of 3.0 × 10^7^–5.0 × 10^7^ cells for each of the selected lines and conditions were collected following dissociation with Accutase and/or centrifugation. Cells were washed with 1XPBS, resuspended in formalin, pelleted at 1000 rpm for 5 min, and fixed overnight at 4 °C. Pellets were gently washed 1× with 70% ethanol to avoid disruption, and centrifuged for 5 min at 1000 rpm. The resulting compact pellet was then embedded in paraffin in a single block to create the CCM. Freshly cut 4–5 µM sections were placed onto slides for IHC analysis.

### Immunohistochemistry

IHC staining was performed at the Tufts Medical Center Histopathology Laboratory (Boston, MA, USA) in a Ventana Medical Systems BenchMark ULTRA automated clinical diagnostic slide stainer (Roche Tissue Diagnostics, Tucson, AZ, USA). For staining with the research-only anti-human ADAM8 rabbit polyclonal antibody LS-B4068 (LifeSpan BioSciences, Seattle, WA, USA), deparaffinized sections (4–5 μm) of formalin-fixed paraffin-embedded (FFPE) cell pellets were subjected to Heat-Induced Epitope Retrieval (HIER) using a Tris–EDTA-based basic buffer, and incubated with primary antibody LS-B4068, or isotype matched control rabbit polyclonal IgG (ab37415, Abcam, Waltham, MA, USA) at 1:50 or 1:100 dilution for 32 min. For detection, a Ventana Medical Systems iVIEW DAB kit (760-091, Roche Tissue Diagnostics, Tucson, AZ, USA) was used. Immunostained slides were counterstained with hematoxylin.

For IHC detection of ADAM8 using ADPs, deparaffinized 4–5 μm thick sections of FFPE cell pellets, patient-derived xenograft (PDX) samples or patient primary tumor samples were subjected to the following optimized conditions: Proteolytic-Induced Epitope Retrieval (PIER) with a 4-min incubation with Protease 2, an alkaline endopeptidase of the serine protease family, plus a signal amplification step using the Ventana Medical Systems Amplification Kit (760-080, Roche Tissue Diagnostics, Tucson, AZ, USA). All other steps within the staining protocol remained the same as described above for LS-B4068. Competition studies were carried out using recombinant human ADAM8 (rHuA8) protein purchased from ACRO Biosystems [amino acids (AA) 17-497, AD8-H5223, Newark, DE, USA] and from R&D Systems (AA1-497, 1031-AD, Minneapolis, MN, USA). ADP2 staining range and linearity were tested using dilutions of 1:50 to 1:120,000. Isotype matched controls were mouse IgG1 (ab18443, Abcam, Waltham, MA, USA) and IgG2b (ab18428 and ab18457, Abcam, Waltham, MA, USA). TNBC PDX tumor samples in TMA and single sample slide format were obtained from StemMed Ltd (Houston, TX, USA) in an anonymous-coded fashion. CCM, PDX and primary breast cancer patient samples were evaluated by a clinical pathologist for sample quality, percent cells staining positive within the cancer component and staining intensity. Initially, a 0 to 3+ staining intensity scale (0: no staining, 1+: low, 2+: medium, 3+: high staining) was used. This was subsequently refined to 0 (no), 0.5 (barely detectable), 1 (faint), 1.5 (moderate), 2 (strong), 2.5 (very strong), and 3 (intense) for higher granularity of the analysis. The manual IHC H-score system, which considers both intensity of staining and percent of cells staining at a particular intensity (H-Score = [(% at < 1) × 0] + [(% at 1+) × 1] + [(% at 2+) × 2] + [(% at 3+) × 3] was adapted for patient sample scoring. As staining was ubiquitous in patient samples, a simplified version of the H-score equal to intensity level × 100% (ranging from 0 to 300) was used and 3 corresponding ADAM8 expression levels: Negative (H-score 0), Low (H-score 50–150) and High (H-score 200–300) were identified.

### Statistical analysis

Remark guidelines for tumor biomarker reporting studies were followed in this study [14]. Data analysis was conducted using SAS software version 9.4 (SAS Institute Inc, Cary, NC, USA). Baseline demographic and disease characteristics were summarized as median (range) for continuous variables and number and percentage for categorical variables. Comparisons between the H-score groups of Negative, Low and High ADAM8 expression were done using Chi-square tests (or Fisher’s Exact test, where appropriate) for categorical variables, or Wilcoxon test for continuous variables. A 10-year Cox proportional hazards model for the HR+ (defined as ER and/or PR positive) and HER2− subtype, with largest sample size, was run to allow for age and race adjusted survival analysis, along with adjusted survival curve. Hazard Ratios with 95% confidence intervals (CI), and parameter estimates and significance with Wald Chi-Square tests were calculated. Survival was calculated as time from surgery until death or censored at time from surgery until last follow-up; if a patient survived beyond 10 years, they were censored at 10 years. If ER, PR or HER2 status, or race were unknown, those patients were not included in subtype specific analyses.

## Results

### Development of an ADP-based IHC assay for the detection and scoring of ADAM8 expression

**Fig. 1:**
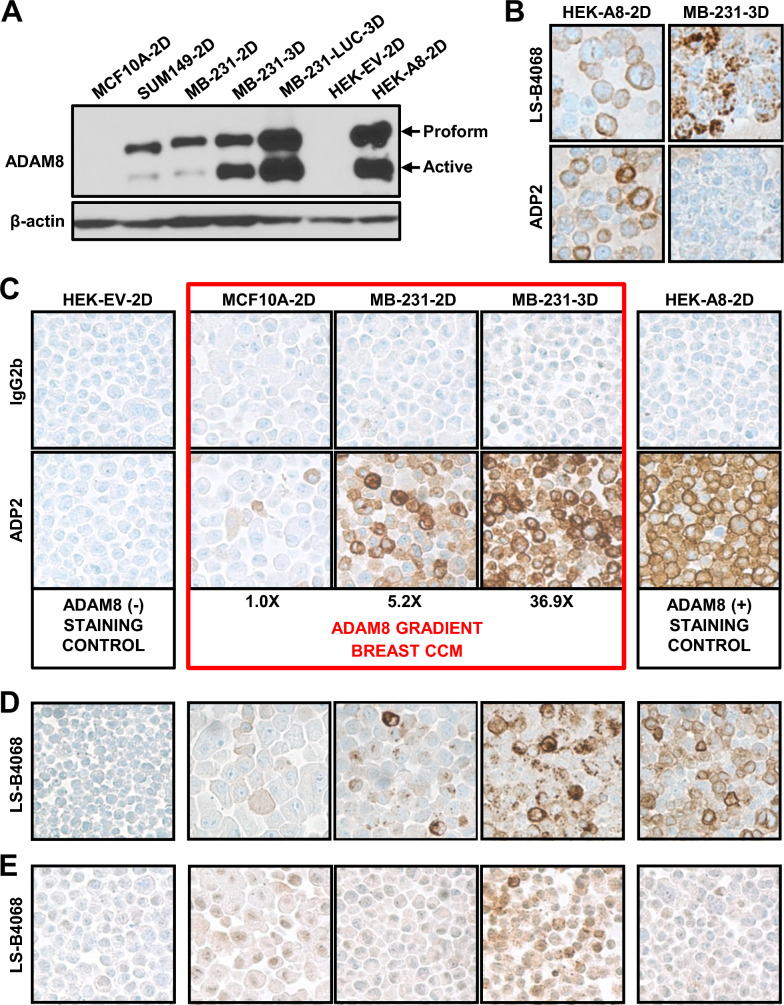
Development of an anti-ADAM8 ADP2 IHC assay and breast CCM. **A** ADAM8 protein levels in breast lines. Protein extracts (30 μg) from breast lines MCF10A, SUM149, MB-231 and MB-231-LUC, and control lines HEK-A8 and HEK-EV, grown in 2D or 3D as indicated, were subjected to immunoblotting with an anti-ADAM8 antibody (LS-C20181), which detected precursor (Proform) and active ADAM8; β-actin was used as loading control. A representative blot of two independent experiments with similar results is shown. To create a breast CCM with a gradient of active ADAM8, MCF10A-2D, MB-231-2D and MB-231-3D were selected; HEK-EV-2D and HEK-A8-2D were included as negative and positive controls, respectively. **B** ADPs did not detect ADAM8 in breast lines under IHC conditions optimized for the commercial anti-human ADAM8 LS-B4068 antibody. CCM slides were subjected to IHC using conditions for the rabbit LS-B4068 antibody and either LS-B4068 or our newly developed ADP antibodies. Representative images of staining in HEK-A8-2D and MB-231-3D cells are shown. Pink color is due to Matrigel used (only) in this early CCM. **C–E** ADP2 under ADP-optimized conditions provides superior ADAM8 detection compared to LS-B4068. CCM IHC staining was performed using the final optimized conditions for the ADP mAbs and either our top diagnostic mAb ADP2 (1:100) or IgG2b (1:100) isotype-matched control for non-specific staining (**C**). The CCM breast lines (red box) displayed ADAM8 levels consistent with those seen in Western blotting (Part **A**). Values given below were determined by densitometry of immunoblots with extracts from independent cultures loaded at various concentrations to ensure a linear range of quantitation, and are presented relative to MCF10A (set to 1.0). IHC of the CCM with LS-B4068 (1:50) and its optimal conditions shows weaker staining than that observed with ADP2 (**D**). IHC of CCM with LS-B4068 (1:100) using ADP conditions shows poorer staining than with its optimal conditions (**E**). Images are at 40× magnification. MB-231, MDA-MB-231

Previously, we isolated a panel of monoclonal antibodies, termed ADPs, with high binding activity to human ADAM8, no cross-reactivity to closely related ADAM family members and strong inhibitory activity, for development into an ADAM8-targeted cancer therapy (manuscript in preparation). Preliminary studies, using FACS analysis of ADAM8-expressing cells demonstrated that 12 of these 18 ADPs were capable of detecting ADAM8 under fixed conditions, raising the possibility that some of them could also be used for diagnosis in IHC. To begin to evaluate their performance in IHC, we first sought to create a staining control and scoring system, containing FFPE pellets of cell lines with a known gradient of increasing ADAM8 levels. To select appropriate lines for this breast CCM, we first analyzed levels of endogenous ADAM8 using immunoblotting. The untransformed mammary epithelial MCF-10A cells and the SUM149 and MDA-MB-231 TNBC lines were selected based on our previous knowledge of ADAM8 protein expression; these were grown for 48–72 h either on plastic (2D) or in suspension culture (3D), which we have shown induces ADAM8 levels [3]. As positive and negative controls, HEK293 cells with either ectopic ADAM8 expression (HEK-A8-2D) or expression of an empty vector DNA (HEK-EV-2D), respectively, were also analyzed. ADAM8 is made as a proform, which can dimerize and autocatalytically clip itself into an active form (Fig. 1A). MCF10A-2D had barely detectable ADAM8 levels, which were more visible with longer film exposures (not shown), whereas SUM149 and MDA-MB-231 cells (labeled MB-231 in all figures) grown in 2D culture expressed moderate levels. Growth of MDA-MB-231 and its more aggressive derivative MDA-MB-231-LUC in 3D culture induced extremely high ADAM8 levels, comparable to those seen in HEK-A8-2D with ectopic ADAM8 expression (Fig. 1A); whereas, HEK-EV-2D cells were negative, as expected. Based on these data, the MCF10A-2D, MDA-MB-231-2D and MDA-MB-231-3D breast lines, and the HEK-EV-2D and HEK-A8-2D controls were selected to create the CCM. To quantify ADAM8 in the CCM breast lines, Western blot analyses were done with independent cultures and extracts at various concentrations to maintain levels within the linear range of the film. Blots were scanned and active ADAM8 levels were normalized to β-actin, which was used as a loading control (Fig. 1A and not shown). MCF10A-2D, MDA-MB-231-2D and MDA-MB-231-3D displayed a stepwise ~ 5- to 7-fold increase in relative active ADAM8 levels, i.e., 1.0, 5.2 and 36.9-fold, respectively (Fig. 1C).

Next, we initiated IHC analysis of the CCM with our ADP antibodies vs the rabbit polyclonal anti-human ADAM8 LS-B4068 antibody, that had been employed in our previous IHC studies of patient samples, using the optimal conditions for LS-B4068 as a starting point [3]. While the ADPs gave good signal with HEK-A8-2D cells, they were unable to recognize endogenous ADAM8 in fixed MDA-MB-231-3D breast cancer cells (Fig. 1B), despite these cells expressing essentially equal amounts of ADAM8 (Fig. 1A). Thus, various steps in the IHC protocol were modified to improve detection of ADAM8 with the ADP mAbs. First, the HIER buffer was modified to a citrate-based acidic buffer, which resulted in lower staining and led us to optimize staining conditions with the original Tris–EDTA-based basic buffer instead. Next, various times of HIER retrieval (i.e., standard: 64 min, shorter: 20 min and longer: 98 min) were tested. Decreased staining of HEK-A8-2D cells was seen with the longer incubation time, suggesting that HIER was inhibiting ADP staining. Next, IHC without HIER epitope retrieval revealed some improvement in staining of endogenous ADAM8 in the breast cell lines, but the relative staining in HEK-A8-2D vs MDA-MB-231-3D was still lower than expected based on the Western blotting. Epitope retrieval with an alkaline endopeptidase of the serine protease family improved accessibility to ADAM8 and staining. Finally, addition of an amplification step resulted in robust diffuse cytoplasmic and membranous staining in the breast vs HEK-A8-2D cells (Fig. 1C) that was now consistent with protein levels (Fig. 1A). MCF10A-2D, MDA-MB-231-2D and MDA-MB-231-3D displayed a substantial increase in the number of cells staining positive within each gradient line consistent with the stepwise increase of five- to sevenfold in relative active ADAM8 levels seen in immunoblotting. A range of staining intensities was observed in all lines (0: no, 1+: low, 2+: medium, 3+: high) (Fig. 1C), consistent with FACS analysis data (not shown), possibly due to changes in expression during cell cycling. These studies suggested MCF-10A-2D, MDA-MB-231-2D, and MDA-MB-231-3D were appropriate cells with a gradient of ADAM8 positivity and intensity for our breast CCM. Using these optimized conditions and the CCM, a comparison of the panel of 18 ADP mAbs identified ADP2 as the top IHC mAb with superior detection of endogenous ADAM8 (comparable to Western blot levels) and signal to noise ratio vs the other ADPs (Fig. 1C and not shown). Notably, ADP2 staining of the CCM was substantially better, under these new conditions (Fig. 1C), than that seen with LS-B4068 under its own optimized conditions (Fig. 1D). LS-B4068 did not work under ADP staining conditions and no improvement in staining was seen with its optimal conditions when an amplification step was added (Fig. 1E and data not shown). Thus, the newly developed ADP2-based IHC assay was found superior to the previously used LS-B4068-based IHC assay for detection of human ADAM8.

### Validation of ADP2-based IHC assay and breast CCM

**Fig. 2:**
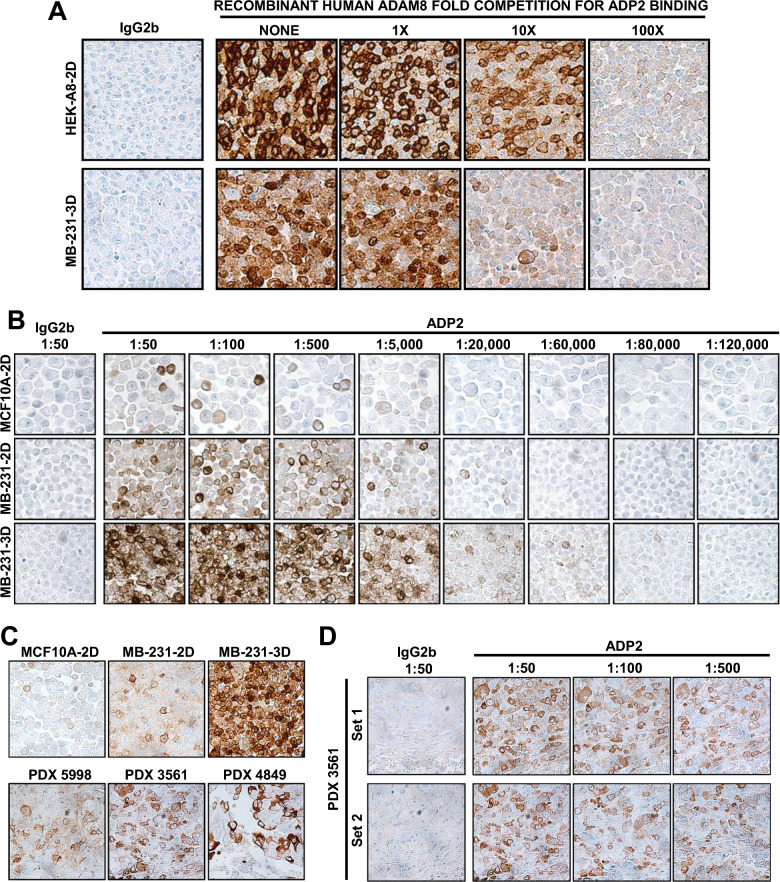
The ADP2 IHC assay detects a range of ADAM8 levels with high specificity and reproducibility. **A** ADP2 staining is specific for ADAM8 in competition analyses. ADP2 was pre-incubated overnight at 4 °C at 1:1000 dilution in the absence or presence of 1×, 10× or 100× molar equivalents of purified recombinant human ADAM8 protein (rHuA8), and used in IHC of HEK-A8-2D and MB-231-3D cells. IgG2b was employed as isotype control. ADP2 staining was reduced in a dose-dependent manner in the presence of rHuA8, demonstrating the assay’s specificity for ADAM8. **B** ADP2 has excellent range and linearity of ADAM8 detection. IHC was performed using slides of the breast CCM and a range of ADP2 dilutions from 1:50 to 1:120,000 under the optimized ADP staining conditions *vs* isotype control IgG2b at 1:50. ADP2 detected both very low and very high levels of ADAM8 in a dose-dependent manner. **C** ADP2 detects ADAM8 in PDX tumor tissue samples at levels that are within the range of the CCM. IHC was performed using ADP2 at dilutions of 1:50, 1:100 and 1:500 *vs* isotype control IgG2b at 1:50 and ADAM8-expressing TNBC PDX samples 5998, 3561, and 4849 *vs* the breast CCM. All 3 PDX samples displayed strong ADAM8 staining that was in the range of that seen in the breast lines of the CCM, indicating the latter is suitable for scoring of more complex tissue samples. Representative images of staining with ADP2 1:50 dilution, which was identified as optimal for tissue sample analysis, are shown. **D** ADP2 reproducibly detects ADAM8 in PDX tumor tissue samples. Consecutively cut slide sets of each PDX were processed on different days (3–4 days apart), and demonstrated equal staining, indicating the reproducibility of the ADP2 IHC assay. Representative images of PDX3561 are shown. Images are at 40× magnification. MB-231, MDA-MB-231

To test the binding specificity of our ADP2 IHC assay, a competition study was performed with increasing doses of the recombinant human ADAM8 (rHuA8), which was used as immunogen for ADP generation. ADP2 was pre-incubated overnight at 4 °C in the presence of 0×, 1×, 10× or 100× molar equivalents of rHuA8, and used in IHC with HEK-A8-2D and MDA-MB-231-3D cells (Fig. 2A). A dose-dependent reduction in staining was seen with rHuA8. Competition with an alternative rHuA8 protein, obtained from a different source, had similar results (not shown). These data confirm ADP2 staining is specific for ADAM8.

We next examined the range and linearity of ADP2 staining of the CCM using dilutions of 1:50 to 1:120,000 (Fig. 2B). Low ADAM8 staining was detected at the 1:50 dilution in MCF10A-2D cells, consistent with the low level seen in longer Western blot exposures discussed above. Staining decreased at 1:5000 and was lost with further dilution. With MDA-MB-231-2D and MDA-MB-231-3D, which express increasing amounts of ADAM8 (Fig. 1A, C), staining was present up to 1:20,000 and 1:80,000, respectively, and disappeared with further dilution (Fig. 2B). Thus, ADP2 detected both extremely low and high levels of ADAM8 in a dose-dependent manner.

Finally, we sought to evaluate the performance of our IHC assay and CCM in PDX tumor tissue samples, ahead of studies in primary patient samples. Thirty TNBC PDX samples were stained with ADP2 (1:100) to identify ADAM8-positive samples (not shown). Three PDX samples (PDX 5998, PDX 3561 and PDX 4849) with significant staining were selected for studies of optimal antibody dilution for tissue staining and for assay reproducibility. For each of these PDX tumors, slides were subjected to IHC with ADP2 (1:50, 1:100 and 1:500). All PDX samples displayed good staining at the 1:50 dilution (Fig. 2C). Similar to breast cancer cells, PDX had diffuse cytoplasmic and membrane staining, with individual cells displaying variable staining intensities. Notably, the highest staining cancer cells within PDX had intensity levels of 2+ to 3+, which were within the range of the CCM (Fig. 2C), confirming selection of appropriate cells. Staining with IgG2b (1:50) was negative, as expected (Fig. 2D and not shown). ADP2 dilution to 1:100 or 1:500 resulted in weaker signal, thus the 1:50 dilution was identified as appropriate for staining of tissue samples (Fig. 2D and not shown). Lastly, staining was comparable in 2 sets run on different days (3–4 days apart), demonstrating assay reproducibility (Fig. 2D and not shown). Thus, our ADP2-based assay and breast CCM were deemed appropriate for staining and scoring of patient samples.

### ADP2-based IHC assay reveals one third of all breast cancers are ADAM8-positive and identifies patients at risk of poor prognosis

**Fig. 3:**
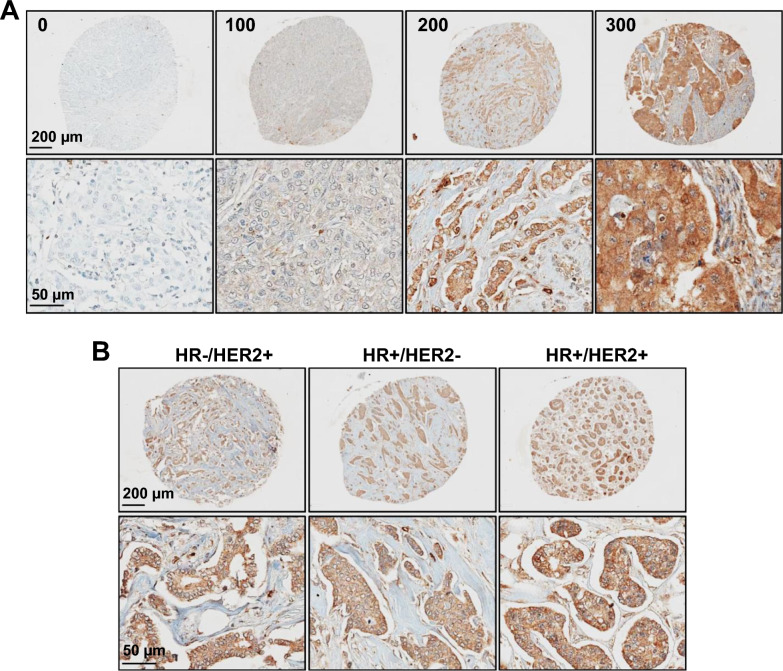
ADAM8 is expressed in all breast cancer subtypes. Freshly cut consecutive TMA slides containing breast cancer patient primary tumor samples were subjected to IHC with ADP2 vs isotype-matched control IgG2b at 1:50 dilutions. H&E staining was used to confirm tissue quality and exclude depleted samples. **A** Representative images of ADP2-stained TNBC samples with increasing ADAM8 expression and associated H-scores are shown. Staining with IgG2b was negative as expected (not shown). Upper panels: whole core images, scale bar: 200 μm; lower panels: magnified images of same samples, scale bar: 50 μm. **B** Representative images of ADP2 IHC staining for ADAM8 in breast cancer patient samples of non-TNBC HR−/HER2+, HR+/HER2− and HR+/HER2+ subtypes. Specimens with high H-scores (e.g., 250–300) are shown. Upper panels: whole core images, scale bar: 200 μm; lower panels: magnified images of same samples, scale bar: 50 μm

Freshly cut, consecutive slides of fourteen TMAs, containing 577 primary breast cancer patient samples were selected for IHC analysis of ADAM8. H&E staining of the TMAs was performed first to evaluate tissue quality and exclude samples that were depleted. This led to the identification of 490 primary breast cancer samples in good condition, including 412 with data on the expression of all 3 standard molecular markers for breast cancer (ER, PR and HER2), for evaluation of ADAM8 expression. Tumor marker status and associated patient characteristics, including age at diagnosis, race, and ethnicity, for the 490 samples can be seen on Table 1. This extensive sample collection allowed us to examine ADAM8 expression for the first time in breast cancer subtypes beyond TNBC (HR−/HER2−), including HR−/HER2+, HR+/HER2− and HR+/HER2+, that we had never examined previously. TMAs and breast cancer CCM were subjected to IHC with ADP2 vs IgG2b as a control for non-specific binding (Fig. 3A, B). Initial evaluation of the patient samples revealed that the intensity of staining was within the range observed in the CCM (not shown), validating its use as a guide for scoring of samples in addition to a process control. Interestingly, patient primary samples when positive, demonstrated diffuse continuous staining (cytoplasmic and membrane) in the cancer component that was ubiquitous and homogeneous in intensity (Fig. 3A, B), unlike ADAM8 staining in cell lines (Fig. 1C) or PDX samples (Fig. 2C, D), which was more heterogeneous. When a sample stained, above 90% of cancer cells were positive and staining was essentially uniform at the same intensity level (Fig. 3A, B). The cancer tissue component of each breast cancer sample mostly fit within a 0, 1+, or 2+ intensity scale vs the CCM with only occasional samples staining at 3+. However, more in-depth examination of the breast cancer samples showed that intermediate intensity levels could be detected. Thus, for added granularity of this early analysis, the intensity scale was expanded to 0 (no), 0.5 (barely detectable), 1 (faint), 1.5 (moderate), 2 (strong), 2.5 (very strong), and 3 (intense). Given the nearly ubiquitous expression, the values for percent positive cells were rounded to 100. This allowed us to adapt the standard manual IHC H-score system, which takes into consideration both intensity and % of cells at a particular intensity (H-Score = [(% at < 1) × 0] + [(% at 1+) × 1] + [(% at 2+) × 2] + [(% at 3+) × 3] and establish a simplified version. An H-score for each sample equal to intensity level × 100% (ranging from 0 to 300) and corresponding ADAM8 expression levels: Negative (H-score 0), Low (H-score 50–150) and High (H-score 200–300) were determined (Fig. 3A). Samples with focal staining (individual cells or cell clusters representing < 1% of the cancer component) were rare and considered negative. A summary of ADAM8 IHC results for the overall patient population (ALL) and each breast cancer subtype can be found on Table 2. Of 61 TNBCs analyzed, 39 were negative, 7 low and 15 high for ADAM8 (Table 2 and Fig. 3A). A total of 36.1% (22/61) of primary TNBC patient tumors stained ADAM8-positive, which is consistent with the 34.0% seen in our early studies of ADAM8 expression in TNBC using the LS-B4068 rabbit anti-ADAM8 antibody [3].

**Table 1:** Summary of patient demographic and disease characteristics

Variable	Total	ADAM8 H-SCORE category			*P-*value
	Population	High	Low	Negative	
	N = 490	N = 115	N = 51	N = 324	
H-score	0.0 (0.0, 300.0)	200.0 (200.0, 300.0)	100.0 (50.0, 150.0)	0.0 (0.0, 0.0)	< 0.0001
Age at diagnosis	59.5 (28.5, 94.8)	67.3 (31.0, 94.8)	58.3 (34.5, 83.7)	58.0 (28.5, 93.2)	< 0.0001
HER2 status					0.1069
Negative	393 (80.2%)	86 (74.8%)	46 (90.2%)	261 (80.6%)	
Positive	57 (11.6%)	14 (12.2%)	3 (5.9%)	40 (12.3%)	
Unknown	40 (8.2%)	15 (13.0%)	2 (3.9%)	23 (7.1%)	
ER status					0.7491
Negative	107 (22.0%)	28 (24.3%)	12 (23.5%)	67 (20.9%)	
Positive	362 (74.3%)	81 (70.4%)	37 (72.5%)	244 (76.0%)	
Unknown	18 (3.7%)	6 (5.2%)	2 (3.9%)	10 (3.1%)	
PR status					0.2145
Negative	160 (32.7%)	44 (38.3%)	13 (25.5%)	103 (31.9%)	
Positive	293 (59.9%)	65 (56.5%)	31 (60.8%)	197 (61.0%)	
Unknown	36 (7.4%)	6 (5.2%)	7 (13.7%)	23 (7.1%)	
Race					0.9142
Asian	2 (0.4%)			2 (0.6%)	
Black/African American	108 (22.0%)	29 (25.2%)	11 (21.6%)	68 (21.0%)	
Other	1 (0.2%)			1 (0.3%)	
Unknown	2 (0.4%)	1 (0.9%)		1 (0.3%)	
White	377 (76.9%)	85 (73.9%)	40 (78.4%)	252 (77.8%)	
Ethnicity					0.0007
Hispanic or Latino	4 (0.8%)	1 (0.9%)	1 (2.0%)	2 (0.6%)	
Non-Hispanic	364 (74.3%)	100 (87.0%)	42 (82.4%)	222 (68.5%)	
Unknown	122 (24.9%)	14 (12.2%)	8 (15.7%)	100 (30.9%)	

Baseline demographic and disease characteristics for the 490 patients whose samples were analyzed for ADAM8 expression are summarized as median (range) for continuous variables, and number and percentage for categorical variables. Following staining and scoring of the TMA samples with the ADP2-based IHC assay, comparisons between the Negative, Low and High ADAM8 expression groups, defined as patients having tumors with ADAM8 H-scores of 0, 50–150 and 200–300, respectively, were done using Chi-square test (or Fisher’s Exact test, where appropriate) for categorical variables, or Wilcoxon test for continuous variables. Analysis was conducted using SAS 9.4 software. P-values are as indicated. N = number of patients

**Table 2:** ADAM8 is expressed in a third of all breast cancers

ADAM8 level	Subtype	ALL			TNBC			HR−/HER2+			HR+/HER2−			HR+/HER2+		
	H-score	n	%		n	%		n	%		n	%		n	%	
Negative	0	324	66.1		39	63.9		16	72.7		201	66.8		19	67.9	
Low	50–150	51	10.4	33.9	7	11.5	36.1	0	0.0	27.3	34	11.3	33.2	1	3.6	32.1
High	200–300	115	23.5		15	24.6		6	27.3		66	21.9		8	28.6	
	Total	490			61			22			301			28		

The results of the ADP2 IHC analysis for ADAM8 in the breast cancer TMAs are summarized in a table format. The number (n) of tumors with Negative, Low or High ADAM8 expression levels, defined as H-scores of 0, 50–150 and 200–300, respectively, as well as corresponding percentages (%), are presented for the 490 total patient samples analyzed (ALL), as well as for each individual breast cancer subtype

**Fig. 4:**
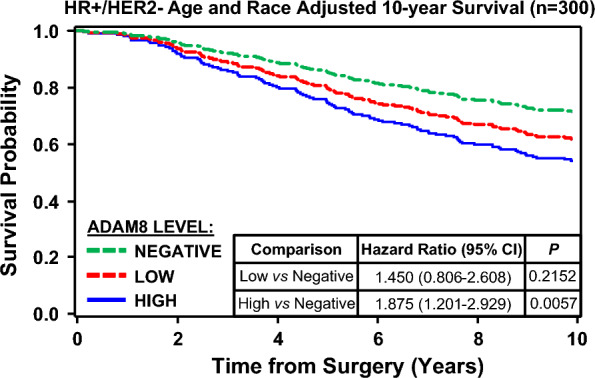
High ADAM8 expression identifies a group of HR+/HER2− breast cancer patients with poor survival. A 10-year Cox proportional hazards model for patients with the HR+/HER2− breast cancer subtype (n = 300 patients) was run using SAS 9.4 software to allow for age and race adjusted survival analysis, along with an adjusted survival curve based on ADAM8 expression levels from the ADP2 IHC assay. Survival was calculated as time from surgery until death or censored at time from surgery until last follow-up; if a patient survived beyond 10 years, they were censored at 10 years. Hazard Ratios with 95% confidence intervals (CI) and significance values of the parameter estimates using Wald’s Chi-Square test are presented. H-scores of 200 to 300, obtained through the ADP2 IHC assay, identified a group of patients at risk of poor outcome

Analysis of ADAM8 expression in breast cancer was extended beyond TNBC to HR−/HER2+ (n = 22), HR+/HER2− (n = 301) and HR+/HER2+ (n = 28) tumors (Table 2 and Fig. 3B). For the first time, these non-TNBC breast cancer samples were similarly found positive for ADAM8: 27.3%, 33.2% and 32.1% for HR−/HER2+, HR+/HER2− and HR+/HER2+ patient samples, respectively (Table 2 and Fig. 3B). Again, when a sample was positive, membrane and cytoplasmic ADAM8 staining was seen that was ubiquitous and homogeneous in intensity. Although the numbers are small, ADAM8 was high in all HR−/HER2+ (6/6) and most HR+/HER2+ (8/9) samples with detectable ADAM8 levels (Table 2). Interestingly, patients with High ADAM8 expression on their tumors were ~ 9 years older at time of diagnosis than those with tumors that were in the Low or Negative categories of ADAM8 expression (Table 1). To determine the significance of ADAM8 expression, a 10-year age and race adjusted Cox proportional hazards model was performed on data from patients with the HR+/HER2− subtype, with largest sample size. High ADAM8 expression, defined as an H-score of 200 to 300, obtained through the ADP2 IHC assay, identified a subset of patients at risk of poor survival (defined as time from surgery until death or censored at last follow-up) (Fig. 4). Thus, ~ 30% of all breast cancer patients have ADAM8-positive tumors and could potentially benefit from an ADAM8-targeted therapy. Furthermore, our data suggest that the ADP2-based IHC assay may have a prognostic value in addition to its original diagnostic intent.

## Discussion

Here, we demonstrate for the first time that ADAM8, a critical driver of tumor growth and spread, is expressed in about one third of all breast tumors, extending our published findings on TNBC to HR+ and HER2+ breast cancer. Furthermore, we show that high ADAM8 levels predict poor outcome in the most common HR+/HER2− subtype. In our earlier work, we focused on TNBC and used a commercially available, research-grade rabbit polyclonal anti-human ADAM8 antibody in IHC studies. Approximately 1/3 of primary TNBCs (17/50 samples = 34.0%) stained positive for ADAM8 [3]. The findings presented here, obtained with our newly developed, highly characterized IHC assay confirm expression of this protein in about one third of TNBCs (22/61 = 36.1%). The analysis also revealed a similar ADAM8 positivity rate (~ 30%) in all other non-TNBC breast cancer subtypes, including HR−/HER2+ (6/22 = 27.3%), HR+/HER2− (100/301 = 33.2%) and HR+/HER2+ (9/28 = 32.1%), that had not been examined previously. Overall, 33.9% (166/490) of all breast cancers expressed ADAM8. Previously, we showed that high *ADAM8* mRNA levels correlate with poor breast cancer patient prognosis [3]. Consistently, here the ADP2 IHC assay and its scoring system reveal a subpopulation of HR+/HER2− patients with high ADAM8 protein levels at risk of poor survival in a 10-year age and race adjusted Cox proportional hazards model. Overall, our findings support the potential role for the ADP2 IHC assay in both the identification and prognosis of patients with ADAM8-positive breast cancers.

While research publications have employed various commercial IHC antibodies to evaluate ADAM8 levels in cancer, these antibodies cannot be used in the clinic due to their lack of characterization (i.e., studies on target specificity, linearity and range of detection). In addition, many are polyclonal rabbit antibodies, including LS-B4068 used in our earlier studies, which are hard to maintain from rabbit to rabbit. Our results provide strong preclinical validation for the use of the ADP2 IHC mAb, our staining protocol and breast CCM for the detection and scoring of ADAM8 in patient samples. The specificity of ADP2 binding to ADAM8 in IHC was confirmed using HEK293 cells expressing ADAM8 vs a control empty vector DNA (Fig. 1C), and competition with either the specific immunogen used to prepare the ADPs or a similar rHuA8 containing the ADAM8 ADP2 binding epitope (Fig. 2A and data not shown). The lack of ADP2 binding to closely related ADAM8 family members ADAM9, ADAM12 and ADAM15 was confirmed during the antibody isolation process in an indirect ELISA using purified recombinant proteins (manuscript in preparation). Furthermore, FACS and IHC analyses of HEK293 cells ectopically expressing ADAM33 confirmed ADP2 lack of cross-reactivity to this closely related family member for which recombinant proteins were not available. ADP2 staining was linear over a wide range of antibody dilutions (Fig. 2B), and reproducible in the more complex PDX tissue samples (Fig. 2D). The levels of active ADAM8 in Western blot analysis of the various breast lines grown under 2D or 3D conditions used in the CCM were commensurate with the extent and intensity of the IHC staining seen in each gradient line (Fig. 1A, C). The range of intensities within the CCM was consistent with that seen in PDX tumors (Fig. 2C) and patient primary tumors (Fig. 3), supporting its use not only as a staining control but also as a scoring system. Together, these findings indicate the suitability of the assay to accurately evaluate patient samples with varied levels of ADAM8 expression. Of note, the ADP2 mAb is stably produced in hybridoma cells, which will ensure a consistent long-term supply in the clinical setting.

According to the American Society of Clinical Oncology (ASCO), the number of women who have died of breast cancer in the United States from 1989 to 2019 has decreased by 42% in large measure due to earlier detection and significant treatment improvements. This translated into the prevention of more than 431,800 breast cancer deaths during that period. However, breast cancer is still the most common cause of cancer death of women worldwide. Thus, additional personalized interventions are urgently needed. Previously, we validated the ADAM8 cell surface protein as a target for treatment of a subset of TNBCs and began development of an antibody-based targeted therapy for this indication (manuscript in preparation) [3]. Our current findings significantly expand the potential patient population, suggesting that diagnosis and subsequent therapeutic inhibition of ADAM8-positive tumors may have much broader implications for breast cancer patients than originally anticipated. Our data demonstrate evaluation of tumors for the presence of ADAM8 may also be useful to stratify breast cancer patients into high-risk vs low-risk prognostic groups. This information could guide the selection of more aggressive treatment regimens when needed, sparing patients at lower risk unnecessarily toxic regimens. In the future, as part of the continued development of this assay, use of digital image analysis will be explored for enhanced assay reproducibility and scalability.

Beyond breast cancer, ADAM8 has also been detected in several other aggressive solid tumors, including lung, liver, pancreas, stomach, colon, bone, and head and neck [4–10]. For all of these cancers, ADAM8 expression was found to be unfavorable. For example, in gastric cancers, the 4th leading cancer-related cause of death globally (WHO), two studies reported higher levels of *ADAM8* mRNA in tumor vs adjacent normal tissue; furthermore, high ADAM8 expression was identified as an independent predictor of poor prognosis, correlating with shorter survival at 3-years (52% vs 82%) and 5-years (31% vs 67%) post-diagnosis [5, 15]. Similarly, elevated ADAM8 protein levels in Hepatocellular Carcinomas (HCC) were closely associated with tumor size (P = 0.007) and metastasis (P = 0.003), and higher tumor stage (P = 0.006) [16]. A clinical grade ADP2 ADAM8 IHC assay would allow for in depth characterization of ADAM8 expression in these multiple indications, providing oncologists with valuable information to facilitate treatment management decisions and improve outcomes.

## Conclusions

Our studies show ADAM8 is widely expressed in breast cancer and provide support for both a diagnostic and prognostic value of the ADP2 IHC assay developed here. As ADAM8 has been implicated in multiple solid malignancies, including those of the breast, lung, liver, pancreas, stomach, colon, bone, and head and neck, continued development of a clinical assay may have broad impact on cancer management.

## Data Availability

The dataset generated and/or analyzed during the current study is not publicly available but may be available from the corresponding author upon reasonable request.

## Abbreviations

AA: Amino acid
ADAM: A disintegrin and metalloprotease
ASCO: American Society of Clinical Oncology
CCM: Control cell line microarray
CI: Confidence intervals
DI: Disintegrin
DFS: Disease-free survival
ER: Estrogen Receptor-α
FFPE: Formalin-fixed paraffin-embedded
FISH: Fluorescence in situ hybridization
HCC: Hepatocellular carcinomas
HEK-A8: HEK-293 cells expressing human ADAM8
HEK-EV: HEK-293 cells transfected with a control empty vector DNA
HER2: Human Epidermal Growth Factor Receptor-2
HIER: Heat-induced epitope retrieval
HR: Hormone Receptor
IHC: Immunohistochemistry
IRB: Institutional Review Board
mAb: Monoclonal antibody
MB-231: MDA-MB-231 cells
MP: Metalloproteinase
OS: Overall survival
PDX: Patient derived xenograft
PIER: Proteolytic-induced epitope retrieval
PR: Progesterone Receptor
rHuA8: Recombinant human ADAM8
RIPA: Radioimmunoprecipitation assay buffer
SERM: Selective estrogen receptor modulator
TMA: Tissue microarray
TNBC: Triple-negative breast cancer
WCE: Whole cell extract
